# An evaluation of the emergency care training workshops in the province of KwaZulu-Natal, South Africa

**DOI:** 10.4102/phcfm.v9i1.1283

**Published:** 2017-03-30

**Authors:** Mergan Naidoo

**Affiliations:** 1 Department of Family Medicine, School of Nursing and Public Health, University of KwaZulu-Natal, South Africa

## Abstract

**Background:**

Emergency care in South Africa is both complex and complicated which is further compromised by inadequately trained healthcare workers. Academic disciplines at the University of KwaZulu-Natal have run emergency care workshops for doctors and nurses providing primary emergency care, in the province for the last 14 years. This delivery of such training has evolved over time.

**Objectives:**

The aim of this study was to evaluate the feedback and knowledge of participants attending the last nine workshops.

**Methods:**

An evaluation questionnaire asked participants to assess the workshops held in the province and to rate their perceived improvement in knowledge. A multiple choice questionnaire was conducted in the last few workshops and was administered pre- and post-workshop. The data were extracted onto an Excel spreadsheet and analysed in Stata version 13. Outcome measures were generated using percentages. A paired *t*-test was used to compare knowledge scores. Open-ended questions were also used to identify areas for future improvement.

**Results:**

The majority (89.4%) of the participants worked in the primary emergency care setting. All participants found the quality of training, the facilitators and the training material good or excellent. Participants’ perceived improvement in knowledge and skills and the objective measure of knowledge improved significantly (*p* < 0.001).

**Conclusion:**

Emergency care education using a combination of inter-professional simulation and lecture-based teaching has the potential of contributing towards better educational outputs in both undergraduate and postgraduate curricula.

## Background

In the province of KwaZulu-Natal (KZN), South Africa, there are 41 district hospitals, 11 regional hospitals and 3 tertiary hospitals that are expected to provide emergency medicine (EM) services to patients accessing care in the public sector.^1^ However, in 2016 only two district and three regional hospitals had emergency care specialists employed who were providing an emergency service. The other hospitals rely on medical officers and/or medical interns to provide the service with the assistance of emergency care nurse practitioners. Many of the staff do not have any formal emergency care training despite having to deal with a complex burden of disease.^2^ Emergency centres (ECs) in KZN are often faced with having to deal with a large burden of HIV patients, which could comprise up to 50% of all patients seen.^3^ These patients often present with complex medical problems affecting multiple organ systems and many need admission for stabilisation and definitive care.^3^ The combination of complex clinical presentations coupled with inadequately trained staff who provide services in most of the district and regional hospitals in KZN has the potential to compromise the quality of patient care.

Since 2002, the Department of Family Medicine at the University of KwaZulu-Natal (UKZN) has had a programme aimed at improving the skills of doctors and nurses providing primary emergency care in the province. From 2002 until 2011, the programme consisted of lectures followed by emergency skills training in the clinical skills laboratory at UKZN. However, in 2011 clinicians from the disciplines of Family Medicine, Emergency Medicine, Nursing, Paediatrics and Trauma Surgery collaborated to put together a modular based programme using a combination of short PowerPoint presentations and simulation-based training using low-fidelity methods because of the resource constraints that exist in the public healthcare environment. The idea behind the training was to identify and train two medical practitioners and two EC nurses based at hospitals and community healthcare centres (CHCs) providing primary emergency care. This method of teaching has been evaluated as having a moderate quality of evidence by the World Health Organization (WHO) which strongly recommends that simulation-based teaching be introduced into health worker curricula.^4^ The WHO guideline further recommends that healthcare worker (HCW) education be provided using an inter-professional approach especially in resource-limited settings as this allows for more HCWs to be trained.^4^

Both these training methods were employed when developing the 10 modules for the 2-day programme. The 2-day programme was developed in 2011 after a group of three family physicians, three emergency care physicians, a surgeon and a nurse reviewed the existing programme and decided to focus on 10 core areas that make up the bulk of morbidity presenting to emergency departments in KZN. Different members of the team were asked to develop the core PowerPoint presentation lasting between 20 and 45 minutes and also develop the skills and simulation scenarios with checklists. The programme was developed along similar lines as the ESMOE (Essential Steps in the Management of Obstetric Emergencies) programme.^5^ Experts then presented their modules and peer review of the content and resources was provided by the group. The programme consisted on the following 10 modules: resuscitation and triage, trauma, common cardiac emergencies, common medical emergencies, sexual assault and ethics in the EC, an approach to the patient with a depressed level of consciousness, common paediatric emergencies, an approach to common poisons and toxins, HIV emergencies and interpreting common radiological investigations in the EC. Skills taught in the programme included resuscitation skills, airway management skills, managing unstable cardiac arrhythmias and immobilising a patient with a cervical spine injury. The South African Triage Scale was also introduced to HCWs and the training was reinforced by asking participants to triage simulated cases presented during small group discussions.^6^ Emergency simulations using case scenarios were used to facilitate learning in most of the modules. This involved dividing participants into small groups and then providing these groups with a scenario that they needed to manage using an inter-professional approach. The group was provided with feedback on the ‘simulated patient’ as they progressed. After the simulation was completed, reflection, peer feedback and facilitator feedback occurred. A scoring checklist was used in the simulations. In some instances, a volunteer from the participants was used as the simulated patient. The programme was then implemented in a decentralised fashion in the various districts in KZN and the manikins and equipment were loaned from the clinical skills laboratory at UKZN. EM Workshops were conducted in the following districts, viz. Ugu, Uthungulu, Umgungundlovu, Umkhanyakude, Zululand, Amajuba, iLembe and eThekwini between 2012 and 2016. Training initially took place in hospitals; however, this was found to be problematic as HCWs were constantly called out of the workshop to attend to problems in the hospital so the venues were changed to external venues in 2014.

The aim of this study was to evaluate the feedback of participants attending the last nine workshops and to assess the written test administered pre- and post-workshops. To the author’s best knowledge, no such evaluation of workshops has been provided to HCWs in the primary emergency care setting in South Africa.

## Methods

Participants who were present for the entire workshop and who agreed to participate in the evaluation completed an evaluation questionnaire at the end of each workshop from 2013. Participants consisted of doctors and nurses providing emergency care at tertiary, regional and district hospitals as well as CHCs and primary healthcare clinics (PHCs). The feedback was used to improve the content of subsequent courses. Since 2015, a pre- and post-course knowledge test in the form of a multiple choice questionnaire (MCQ) was administered. The evaluation questionnaire was based on the ESMOE evaluation tool and the MCQ test was developed by the course coordinator based on the content of the module.

The evaluation questionnaire asked participants to assess the quality of training, the trainers, the course material, their confidence in using the course material in running similar courses in their own institutions, the content of the course and a perceived improvement in knowledge and skills pre- and post-course attendance. The MCQ consisted of 10 questions based on each module of the course. The questions were in a true and false format. A retrospective review of the responses of the evaluation questionnaires and the results of pre- and post-test written examination was performed.

The data were extracted onto an Excel spreadsheet and analysed in Stata version 13. Outcome measures generated included means, standard deviations and percentages. A paired *t*-test was used to compare perceived improvement in knowledge based on a rating scale and the knowledge scores from the MCQ test pre- and post-workshop. Significance was set at the 5% level, that is, *p* < 0.05. Open-ended questions in the evaluation questionnaire were analysed and the commonest responses were thematically presented based on the responses received. Not all participants responded to these questions.

## Results

Eleven 2-day workshops were held in various districts in KZN and 321 HCWs were trained. Between 61.56% (*n* = 135) and 86.6% (*n* = 278) completed various elements of the evaluation questionnaire. Seventy-two percent (*n* = 135) out of an eligible 187 participants completed the MCQ test. Some of the workshops involved HCWs from more than one district. Table 1 presents an outline of the various workshops held in the province over the last 5 years.

**TABLE 1:** Outline of the emergency medicine training workshops done in KZN.

Date	Participants		Venue	Districts covered	Course evaluation done	Pre-post-test

	Doctors	Nurses				
25–27 June 2012	20	8	Skills Laboratory Durban	eThekwini	No	No
26–28 Sept 2012	7	14	Murchison Mission	Ugu	No	No
10–12 June 2013	22	0	Northdale Hospital	uMgungundlovu	Yes	No
23–24 May 2014	24	2	Ghost Mountain Inn	Umkhanyakude	Yes	No
1–2 August 2014	11	8	Tsogo Sun Ulundi	Zululand	Yes	No
21–22 November 2014	10	8	Tsogo Sun Newcastle	Amajuba/Umzinyathi	Yes	No
20–21 February 2015	25	36	Skills Lab DURBAN	eThekwini/iLembe	Yes	Yes
26–27 June 2015	35	21	Tsogo Sun Pietermaritzburg	uMgungundlovu/Harry Gwala/Umzinyathi	Yes	Yes
18–19 March 2016	14	21	Salt Rock Hotel	Ilembe	Yes	Yes
6–7 May 2016	9	9	St Michaels Beach Hotel	Ugu	Yes	Yes
22–23 July 2016	7	10	Richards Bay Protea Hotel	Uthungulu	Yes	Yes

**Total**	**184**	**137**	**11 venues**	**11 districts**	**-**	**-**

HCWs consisted of emergency room nurses and doctors and clinical or nursing managers. The majority of the staff (65%) worked at district hospitals, 11.5% of staff were based at CHCs and PHCs and the remainder of the staff worked at regional or tertiary hospitals and the district office. Participation from HCWs based in rural districts were poor and this was often ascribed to poor staffing and long distances that needed to be travelled to get to the workshop. The majority (89.4%) of the participants who completed the questionnaire worked in the primary emergency care setting as the regional hospitals were all combination regional-district hospitals that provided a primary emergency care service. HCWs evaluated the programme using a scale between 1 and 5, with a score of 1 rated as poor, 2 as fair, 3 as average, 4 as good and 5 rated as excellent. Table 2 depicts the mean scores.

**TABLE 2:** Satisfaction with the training programme – Pooled analysis.

Variable	*N*	Mean	s.d.
What is your overall impression of this training programme?	235	4.64	0.56
How satisfied were you with the venue?	278	4.57	0.65
How satisfied were you with the organisation or the training programme?	278	4.58	0.62
How satisfied were you with the training material?	236	4.44	0.96
How satisfied were you with the skills demonstrations/simulations?	259	4.53	0.67
How satisfied were you with the facilitators?	259	4.61	0.64

*N*, Number of participants who filled in this section of the evaluation questionnaire; s.d., standard deviation.

Most participants found the quality of training, the facilitators and the training material good or excellent. Participants expressed a willingness to use their new found knowledge by implementing changes in their own institutions either by training others or by changing their current practice. Responses to questions on whether they would train others in their institution revealed that 92.64% (*N* = 231) found the course comprehensive enough to provide them with the confidence to train others at their institutions and 93.51% (*N* = 231) stated that they will be doing EM training at their own institutions.

Participants were asked to assess their improvement in knowledge and skills after attending the workshop. A paired *t*-test was used to analyse responses based on a scale of 0 (I have no knowledge or skill) to 5 (I can do this and I can teach others). Results are shown in Table 3.

**TABLE 3:** Change in knowledge and skills using a paired *t*-test: Pooled analysis.

Module	Self-assessment	*N*	Mean	s.d.
Resuscitation and triage	Pre	211	2.71	1.25
	Post	211	4.27	0.88
Trauma	Pre	211	2.79	1.21
	Post	211	4.02	1.03
Cardiac emergencies	Pre	207	2.60	1.22
	Post	207	3.89	0.96
Medical emergencies	Pre	207	2.85	1.18
	Post	207	3.96	1.04
Ethics - sexual assault	Pre	197	2.53	1.37
	Post	197	3.75	1.18
Unconscious patient	Pre	211	2.89	1.22
	Post	211	4.05	1.03
Toxicology	Pre	207	2.56	1.19
	Post	207	3.83	1.04
Paediatrics	Pre	208	2.73	1.16
	Post	208	3.86	1.03
HIV emergencies	Pre	212	2.64	1.20
	Post	212	3.86	1.03
Radiology	Pre	211	2.42	1.37
	Post	211	3.85	2.18

*N*, Number of participants who filled in this section of the evaluation questionnaire; s.d., standard deviation.

The pooled results from all the workshops indicate that participants’ felt that their knowledge and skills improved significantly across all modules (*p* < 0.001). The mean difference across all modules pre-workshop compared to post-workshop was -1.22 (95% confidence interval: -1.45; -0.99), which was statistically significant. The results obtained via an MCQ administered at the last five workshops are listed in Table 4.

**TABLE 4:** Analysis of pre- and post-test results of the multiple choice question examination.

Variable	*N*	Mean	s.e.m.	s.d.	95% Confidence interval	
Entire group						
Pre-test	135	4.02	0.14	1.63	3.75	4.30
Post-test	135	6.12	0.14	1.64	5.84	6.40
Nurses						
Pre-test	70	4.3	0.22	1.82	3.86	4.73
Post-test	70	6.63	0.19	1.58	6.25	7.01
Doctors						
Pre-test	65	3.72	0.17	1.34	3.39	4.05
Post-test	65	5.57	0.19	1.53	5.18	5.95

s.e.m., standard error of the mean; *N*, Number of participants who filled in this section of the evaluation questionnaire; s.d., standard deviation.

Knowledge improved significantly post-workshops for both doctors and nurses with the mean score of nurses being higher than the mean score for doctors. The MCQ examination had 10 questions and the maximum mark was 10.

Participants were asked to list any suggestions they may have on how their knowledge and skills could be improved. The most common suggestions were (number of responses in brackets) the following:

- Thank you, well done, I gained lots of knowledge. (94)
- Simulations should be done in the workplace to reinforce knowledge and skills. (42)
- Simulations should be done weekly. (20)
- Please provide accommodation. (15)
- These workshops should be conducted at each hospital. (13)
- Please conduct the workshop more often. (13)
- Develop an emergency care workshop for PHC nurses. (13)
- Add the skill of sub-cutaneous pacing. (10)
- More time is needed for this workshop. (10)
- Add a section on dermatology. (9)

## Ethical consideration

Ethical approval to publish the findings of this evaluation was obtained from the UKZN Biomedical Research Ethics Committee (Reference BE 427/15). Funding for the training was obtained from the UKZN Medical Education Partnership Initiative (MEPI).

## Discussion

The main study findings were participants rating the overall workshop as good or excellent and a significant improvement in knowledge and skills.

The bulk of the participants were from institutions which provided primary emergency care and this may have been achieved because of the decentralised programme. Although the project used a decentralised district-based model for training, the participation from rural districts was poor because of distances that needed to be travelled to get to the venue as well as poor staffing levels at rural district hospitals. The centralised model of training that is being used by the ESMOE programme in KZN may be a better suited model.^7^ The ESMOE master trainer programme provides accommodation for participants in Durban, but this cost could be defrayed against the cost of venue hire and meal provision at rural sites. This would also assist some participants who requested that accommodation be made available.

The use of low-fidelity simulations was appreciated by all participants in contrast with the perception that simulation-based training is expensive.^8^ Low-fidelity simulation training in this setting required participants to volunteer to be patients and basic emergency equipment was provided for practice of some of the skills. High-fidelity training generally expects the simulation to replicate the real-life scenario to a greater extent and this is done using advanced training manikins that make use of sophisticated computer programmes with/ without the aid of a skilled technician.^9^ Simulation-based training allows for the acquisition of clinical expertise in a non-threatening environment that does not compromise patient safety. Experiential clinical learning in the real clinical environment is the gold standard for learning but comes at a risk to patient safety, whereas simulation-based training allows participants to practice, reflect, get feedback and re-practice until satisfied with their competencies.^8^,^9^ Like many other interventions in medicine, simulation-based training has its roots in the aviation industry which reported a 90% reduction in collisions during adverse weather conditions when a ‘primitive simulator’ was used early in the 20th century.^9^ Anaesthesia and surgery were the first disciplines to use simulation-based training in their curricula, but this has now expanded to many other disciplines of medicine.^9^,^10^,^11^ The EM postgraduate programmes in the United States have in particular seen a significant growth of simulation-based training during the period 2003–2008.^10^ Many of these programmes use high-fidelity simulators^10^ in contrast with the low-cost simulations used in this programme.

The majority of the participants rated the overall workshop as good to excellent with similar sentiments made on the quality of the logistics, the facilitators, the simulations and the resource material provided. These findings are consistent with those of Mugford and colleagues^12^ who found similar results in the primary care setting in Australia. The workshop had its own evolutionary process with feedback from participants being used to improve subsequent courses. Using scenarios based on actual case studies from clinical practice allows one to improve content validity and hopefully make inferences to authentic patient clinical pathways.^13^ Simulation also allows one to develop clinical skills, reflective practice, procedural skills and to develop skills in identifying deficiencies in the process and organisation of clinical care especially when used in the workplace.^13^ Participants were encouraged to use their newly acquired skills in their places of work and many indicated that they will do so. Using the facilitator resources that were provided on their DVDs, they were encouraged to run these simulations in a real environment and identify gaps in terms of resources and process flows, which would then facilitate the development of quality improvement projects.

Participants’ subjective assessment of their perceived knowledge showed significant improvement, which was then ratified when the pre- and post-test questionnaires were administered during the last few workshops. Based on Pratt’s^14^ nurturing perspective of teaching, one needs to acknowledge that effective teaching must respect adult learners’ ‘self-concept and self-efficacy’ in assessing their knowledge and skills. A combination of lecture-based teaching that highlighted key points in very short presentations followed by simulation drills improved the short-term knowledge scores. This is consistent with other studies which found similar results when used to teach toxicology.^15^ Simulation-based training has also been found to improve knowledge retention.^8^,^9^,^11^,^15^ Clinical simulation can easily be adapted for a clinic or emergency room setting. This is a useful tool for maintaining expertise in core emergency skills, which may only be needed on rare occasions in the real world. It is important that the simulation format of introduction, drill execution, personal reflection and then feedback is used to reinforce knowledge and skills. The workshop allowed for a before and after drill to be performed, which allowed participants a practice run after feedback. Simulation would fit into the category of ‘shows how’ if one had to extrapolate this to Millers pyramid of clinical teaching.^16^ The training would satisfy formative training and could easily augment work-integrated learning.^4^ Many participants expressed a willingness to integrate simulation training in the clinical environment and this was encouraged as it has been shown to improve skills in the ESMOE programme in KZN.^7^

The baseline mean and post-test mean knowledge score for nurses was higher than that of doctors and this may be because of the fact that nurses tend to be employed in the public sector for much longer periods than doctors,^17^,^18^ which would have given them much better exposure to experiential learning in the emergency environment.

The inter-professional approach to training not only mimics the real work environment but also contributes towards improved teamwork and collaboration.^11^,^19^ Human simulation using standardised patients who were participants in the workshop worked well in many of the scenarios. Simulations are excellent for improving communication skills and interpersonal and profession skills.^11^ Using role definitions in scenarios further facilitated team work and outcomes in the simulations. It also allowed participants to appreciate the input from their colleagues from other disciplines. In the South African environment, one often finds this in junior doctors working with senior nurses who can provide invaluable input in a stressful emergency care environment.

## Limitations of the study

The workshop content followed an evaluation, analysis and modification throughout the 4 years between 2012 and 2015. In addition, the first few workshops were held at venues within the hospital and this created problems with participation and no formal evaluation was done of these courses. The programme was evaluated by the administrator of the course, and despite being anonymously filled in, one would expect that an element of bias may exist. No long-term assessment of an improvement in knowledge and skills was performed to indicate if the new knowledge would be retained. The impact on work place–based practice and on patient outcomes was also not evaluated.

## Conclusion

Emergency care education using a combination of inter-professional simulation and lecture-based teaching has the potential of contributing towards better educational outputs for nurses and doctors working in the primary emergency care setting. It is recommended that such training be introduced in both undergraduate and postgraduate curricula. A recommendation would be that future workshops be run at centralised venues as these attract better participation and if funds permit, accommodation be provided for participants from remote and rural sites. The recommendation of developing an emergency care workshop specifically designed for PHC nurses has been actioned and is being rolled out in the eThekwini district. A follow-up study is being contemplated to contact participants to establish if the training led to changes in clinical practice.
